# The Intrapericardial Delivery of Extracellular Vesicles from Cardiosphere-Derived Cells Stimulates M2 Polarization during the Acute Phase of Porcine Myocardial Infarction

**DOI:** 10.1007/s12015-019-09926-y

**Published:** 2019-12-21

**Authors:** Esther López, Rebeca Blázquez, Federica Marinaro, Verónica Álvarez, Virginia Blanco, Claudia Báez, Irene González, Ana Abad, Beatriz Moreno, Francisco Miguel Sánchez-Margallo, Verónica Crisóstomo, Javier García Casado

**Affiliations:** 1 Stem Cell Therapy Unit, Jesus Uson Minimally Invasive Surgery Centre, Cáceres, Spain; 2 CIBER de Enfermedades Cardiovasculares (CIBERCV), Madrid, Spain

**Keywords:** Extracellular vesicles, Cardiosphere-derived cells, Acute myocardial infarction, Intrapericardial administration, Inflammation

## Abstract

Acute myocardial infarction triggers a strong inflammatory response in the affected cardiac tissue. New therapeutic tools based on stem cell therapy may modulate the unbalanced inflammation in the damaged cardiac tissue, contributing to the resolution of this pathological condition. The main goal of this study was to analyze the immunomodulatory effects of cardiosphere-derived cells (CDCs) and their extracellular vesicles (EV-CDCs), delivered by intrapericardial administration in a clinically relevant animal model, during the initial pro-inflammatory phase of an induced myocardial infarction. This effect was assessed in peripheral blood and pericardial fluid leukocytes from infarcted animals. Additionally, cardiac functional parameters, troponin I, hematological and biochemical components were also analyzed to characterize myocardial infarction-induced changes, as well as the safety aspects of these procedures. Our preclinical study demonstrated a successful myocardial infarction induction in all animals, without any reported adverse effect related to the intrapericardial administration of CDCs or EV-CDCs. Significant changes were observed in biochemical and immunological parameters after myocardial infarction. The analysis of peripheral blood leukocytes revealed an increase of M2 monocytes in the EV-CDCs group, while no differences were reported in other lymphocyte subsets. Moreover, arginase-1 (M2-differentiation marker) was significantly increased in pericardial fluids 24 h after EV-CDCs administration. In summary, we demonstrate that, in our experimental conditions, intrapericardially administered EV-CDCs have an immunomodulatory effect on monocyte polarization, showing a beneficial effect for counteracting an unbalanced inflammatory reaction in the acute phase of myocardial infarction. These M2 monocytes have been defined as “pro-regenerative cells” with a pro-angiogenic and anti-inflammatory activity.

**Electronic supplementary material:**

The online version of this article (10.1007/s12015-019-09926-y) contains supplementary material, which is available to authorized users.

## Introduction

Cardiac heart failure is one of the main death causes worldwide [1]. In acute myocardial infarction (AMI), the cardiac tissue injury triggers a strong inflammatory process where immune cells and soluble mediators are involved. This inflammatory process has been extensively studied, characterized and sequenced. An initial pro-inflammatory phase occurs at days 1–3 after myocardial infarction, and is followed by an anti-inflammatory phase at days 4–7, to achieve tissue repair [2]. During the pro-inflammatory phase, cell death and tissue injury trigger the release of “danger signals” (i.e. activation of complement cascade, reactive oxygen species production, Toll-like receptor activation), which induce cytokine/chemokine production for the recruitment of leukocytes, such as neutrophils, monocytes, and T cells, to the myocardial infarction area. After myocardial infarction, the bone marrow initiates the activation of hematopoietic cells and leukocyte production which are mobilized into the blood [3].

Nowadays, many different anti-inflammatory therapies, aimed to modulate an adverse and unbalanced inflammation, are under evaluation [4]. Among them, cell therapy has become a valuable tool since stem cells have proved to exert an immunomodulatory effect on different immune mediated diseases [5, 6]. In relation to myocardial infarction, the intracoronary delivery of cardiosphere-derived cells (CDCs) has already been found to attenuate myocardial inflammation in a murine model of autoimmune myocarditis [7], and to reverse inflammation and fibrosis in hypertensive rats [8]. The intramyocardial injection of CDCs in a myocardial porcine model have demonstrated an inverse correlation between doses and engraftment, together with an increase of left ventricular ejection fraction [9]. Few years later, these authors could also demonstrate that cardioprotection was safe and effective using 7.5 × 10^6^ to 10 × 10^6^ allogeneic CDCs [10]. More recently, exosomes secreted by CDCs were evaluated in an acute and chronic porcine myocardial infarction models, demonstrating that intramyocardial administration (but not intracoronary) decreased scarring, and improved cardiac function [11]. These exosomes have also demonstrated an immunomodulatory effect [12], an anti-apoptotic protection under in vitro conditions [13], as well as favorable effects on hearts from aged rats [14].

In stem cell-based therapies, the administration routes are key factors for the treatment success. Our previous studies have demonstrated that the intrapericardial administration of mesenchymal stem/stromal cells (MSCs) in a porcine model of myocardial infarction provided an optimal retention and implantation of MSCs in the infarcted heart [15]. A similar work using CDCs showed that these cells exert changes in pericardial fluid lymphocytes after intrapericardial administration [16]. Based on these observations, here we hypothesize that CDCs and/or their extracellular vesicles (EV-CDCs) may counterbalance an exacerbated inflammatory reaction during the acute phase of myocardial infarction.

For understanding the immunological mechanisms involved in AMI after the therapies, the present study has been carried out in a porcine model. This clinically relevant animal model has been widely accepted by researchers and regulatory agencies as a valuable tool in the evaluation of safety aspects and efficacy of new therapeutic products [17].

Our preclinical study has demonstrated a successful myocardial infarction induction in all animals and no adverse effect was seen for intrapericardial administration of CDCs or EV-CDCs. As expected, significant changes were observed in biochemical and immunological parameters after myocardial infarction. A complete and exhaustive analysis of peripheral blood leukocytes revealed an increase of M2 monocytes at 24 h after EV-CDCs administration, while no differences were reported in other lymphocyte subsets. Moreover, arginase-1 (a classical M2-differentiation marker) was significantly increased in pericardial fluid at 24 h after EV-CDCs administration.

In summary, here we demonstrate that, in our experimental conditions, intrapericardially administered EV-CDCs have an immunomodulatory effect, enhancing M2 monocyte polarization. These M2 monocytes have been defined as “pro-regenerative cells” with a pro-angiogenic and anti-inflammatory activity which may counteract an unbalanced inflammatory reaction in the acute phase of myocardial infarction.

## Methods

### Animals and Experimental Design

**Fig. 1:**
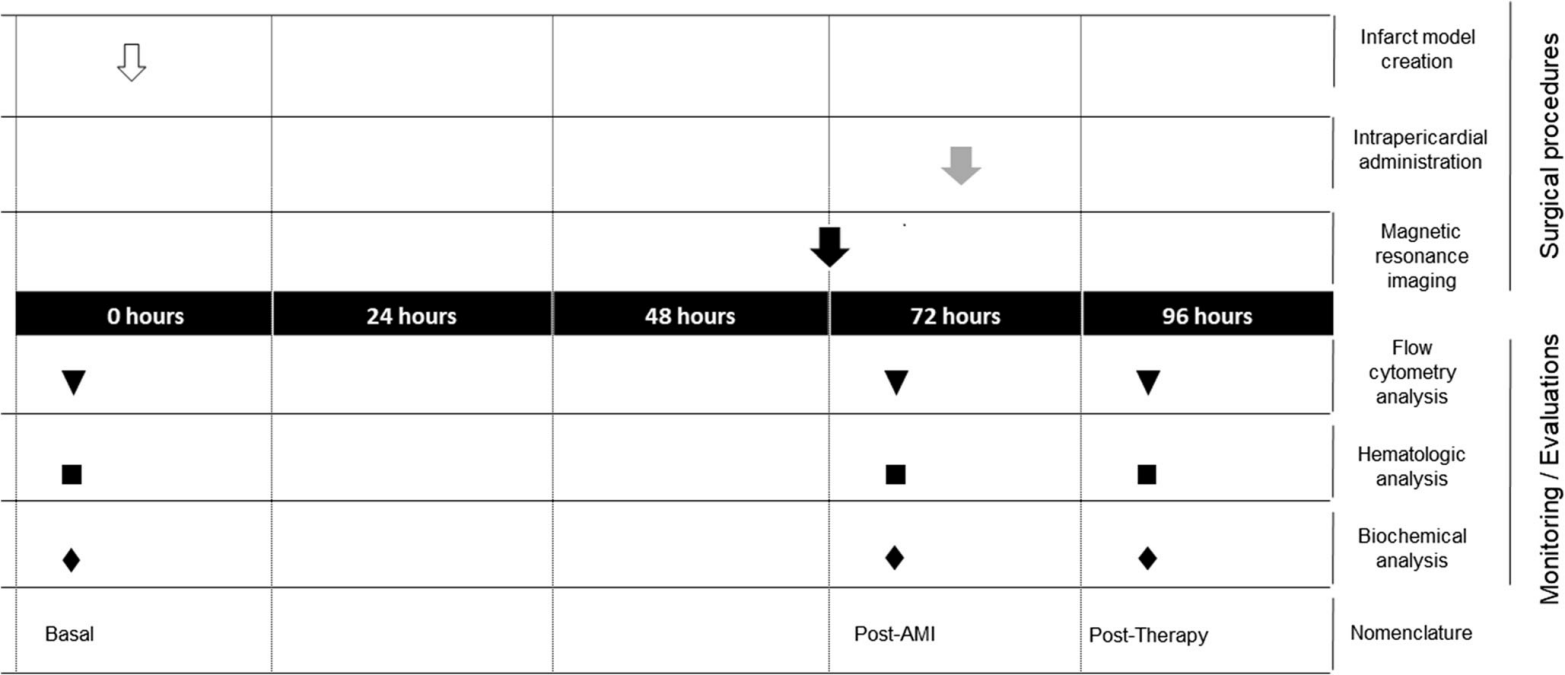
Experimental design. At day 0, infarct model was created (white arrow). At 72 h, magnetic resonance imaging was performed (black arrow). Placebo, EV-CDCs or CDCs were intrapericardially administered at 72 h (grey arrow). Blood samples were collected at day 0 (Basal), 72 h (post-AMI) and 24 h after intrapericardial administration (post-therapy). Blood samples were used for flow cytometry analysis (triangles), hematology (squares) and biochemical analysis (rhombus)

Female Large White pigs (*n* = 18), weighting 36.68 ± 5.18 kg at the beginning of the study, were used for all experimental procedures. Fifteen animals were randomly divided into three groups: Placebo (*n* = 5), CDCs (n = 5), and EV-CDCs (n = 5). Three additional animals were used to evaluate the effect of EV-CDCs on the pericardial fluid leukocytes. The time points for myocardial infarction induction, blood sampling, magnetic resonance imaging, and intrapericardial administration are schematized in Fig. 1. Animals were housed in the animal facility of the Jesús Usón Minimally Invasive Surgery Centre. The final destination of all animals was the euthanasia, performed with an intravenous administration of 2 mmol/kg of KCl, applied under deep anesthesia. The experimental procedures were validated by the Ethics Committee on Animal Experiments of the Jesús Usón Minimally Invasive Surgery Centre, in accordance with the recommendations outlined by the local government (Junta de Extremadura), and the EU Directive 2010/63/EU of the European Parliament on the protection of animals used for scientific purposes.

### Isolation and Characterization of CDCs

CDCs were isolated from cardiac explants of experimental Large White pigs. Briefly, explants were mechanically disaggregated and digested three times with a solution of 0.2% trypsin (Lonza, Basel, Switzerland), and 0.2% collagenase IV (Sigma, St. Louis, MO). The cell culture isolation and in vitro expansion were performed as previously described by our group [16].

### Isolation and Characterization of EV-CDCs

EV-CDCs were obtained from cell lines cultured at a confluence of 80% at passages 12–15. Culture medium (DMEM with 10% Penicillin/Streptomycin, and 10% Fetal Bovine Serum) was replaced by exosome isolation medium (1% insulin-transferrin-selenium in DMEM with 10% Penicillin/Streptomycin). The supernatants were collected at day 4 and centrifuged in two steps: first at 1000 x g for 10 min, and then 5000 x g for 20 min at 4 °C. Supernatants were filtered through a 0.22 μM filter to eliminate dead cells and debris, and ultra-filtered through a 3 kDa MWCO Amicon® Ultra device (Merck-Millipore, MA, USA). EV-CDCs were firstly characterized by Field-Flow Fractionation carried out with a regenerated cellulose membrane (cut off 10 kDa), and with a spacer of 350 mm. Finally, a high throughput proteomic analysis was performed on EV-CDCs using high-resolution liquid chromatography coupled to mass spectrometry-based proteomic analyses, as described in our previous studies [18, 19]. The detected proteins were classified according to the Gene Ontology term GO:0070062 (*Extracellular exosome*). For the intrapericardial administrations, protein quantifications were performed by Bradford assay, and the resulting protein concentration was diluted in Sodium Chloride 0.9% (*w*/*v*) to 1.832 mg/ml. Finally, 9.16 mg of exosomal proteins were intrapericardially administered in 5 mL of Sodium Chloride 0.9%.

### Myocardial Infarction Model Creation

The animal model was created by a closed chest reperfused myocardial infarction technics, as previously described [20]. Briefly, animals were anesthetized and subjected to coronary angiograms in the 40° left anterior oblique projection. The occlusion of blood flow to the distal myocardium was carried out with the insertion of a coronary balloon catheter (typically 3 mm × 8 mm, Ryujin Plus, Terumo, Tokio, Japan) over a 0.014″ coronary guidewire until reach the origin of the first diagonal branch. The balloon was inflated for 90 min and after balloon deflation, coronary patency was evaluated by another angiogram. Animals were monitored during the procedure, evaluating different parameters like blood pressure, electrocardiogram, O_2_ saturation, and end tidal CO_2_. Lidocaine (Lidocaine, Braun Medical, Barcelona, Spain) was perfused during the procedure at a rate of 1 mg/kg/h and heparin (Heparina Rovi 5%, Laboratorios farmacéuticos Rovi, Madrid, Spain) was intravenously injected at 150 UI/kg before of the intervention.

### Cardiac Magnetic Resonance

Cardiac Magnetic Resonance studies (Intera 1.5 T, Philips Medical Systems. Best, The Netherlands) were acquired at 72 h after myocardial infarction. A dedicated cardiac coil was used. Acquisitions were performed in the intrinsic cardiac view: short axis, two- and four-chamber views. For morphological and functional studies, gradient echo cine exams, including the entire left ventricle, were performed during apneas. Scar sizes were computed in short axis delayed enhancement studies performed 5–15 min after contrast administration at a dose of 0.2 mmol/kg of gadolinium (Gadobutrol. Gadovist 1.1 mmol/l, Bayer Schering Pharma AG, Berlin, Germany) with a breath-hold 3D gradient-echo inversion-recovery sequence. Typical inversion time determined with the Look and Locker sequence was 150 to 190 ms to obtain the best myocardial nulling. A slice thickness of 8 mm without gap, a FOV: 330 × 330 × 50, matrix: 224 × 200, flip angle: 15° and TR/TE: 4.9/1.67 were used for imaging. Post-processing was done using the scanner’s own software (IntelliSpace Portal 7.0.2.20700 Philips Medical Systems, Best, The Netherlands) by a blinded researcher. Automatically detected endo and epicardial borders were carefully reviewed, and corrected when necessary to compute left ventricular function in terms of indexed End Diastolic (EDVi) and Systolic (ESVi) volumes and Ejection Fraction (% LVEF). To calculate infarct size, the percentage of left ventricular mass in normal conditions, and in the infarcted myocardium were identified (% Infarct) in short axis delayed enhancement views, with dark areas corresponding to hemorrhage or microvascular obstruction included.

### Intrapericardial Administration

72 h after myocardial infarction induction, animals were pre-medicated with diazepam 0.3 mg/kg and ketamine 10 mg/kg intramuscularly. Anesthesia induction was achieved with 2 mg/kg of propofol and maintained with 1.8%–2% sevofluorane. Auricular vein were catheterized to perfuse normal saline to preserve hydration. During this procedure, animals’ parameters were monitored, as in the previous section.

A total of 5 animals received an intrapericardial injection of 30 × 10^6^ allogeneic CDCs in 5 ml of sodium chloride 0.9% (w/v). Animals treated with EV-CDCs (n = 8) received a total of 9.16 mg EV-CDCs proteins per animal in 5 ml of sodium chloride 0.9% (w/v). The placebo group (n = 5) received 5 ml of sodium chloride 0.9% (w/v). The administration was achieved via mini-thoracotomy and placebo, EV-CDCs, or CDCs were administered using an Abbocath®-T 20G catheter (Hospira, Lake Forest, IL, USA). The incision was closed layer by layer and the animals were let to recover.

### Troponin I Analysis

Blood samples were collected in EDTA tubes, and used for Troponin I analyses at 72 h after myocardial infarction. Concentration of Troponin I was quantified in terms of μg/l by immunoassay (AQT90 Flex, Radiometer Iberica SL, Madrid, Spain).

### Biochemical Analyses

Biochemical analyses of peripheral blood samples were performed in the clinical analyzer Metrolab 2300 (Metrolab S.A., Buenos Aires, Argentina). The following serum parameters were determined: bilirubin, creatinine, glucose, urea, gamma-glutamyl transferase (GGT), glutamic oxaloacetic transaminase (GOT), glutamic pyruvic transaminase (GPT), and total proteins.

### Hematological Analysis

Hematological analyses were assessed in an automatic hematology analyzer (Mindray BC-5300 Vet, Hamburg, Germany). The blood parameters determined were: white blood cell count (WBC), neutrophils, lymphocytes, monocytes, eosinophils, basophils, red blood cell count (RBC), hemoglobin concentration (HGB), hematocrit (HCT), mean corpuscular volume (MCV), mean corpuscular hemoglobin (MCH), mean corpuscular hemoglobin concentration (MCHC), red blood cell distribution width coefficient of variation (RDW-CV), red blood cell distribution width standard deviation (RDW-SD), platelets (PLT), mean platelet volume (MPV), platelet distribution width (PDW), and plateletcrit (PCT).

### Phenotypic Characterization of Peripheral Blood Leukocytes

Peripheral blood leukocytes were isolated by centrifugation over Histopaque-1077 (Sigma, St. Louis, MO), and washed twice with PBS. Cells were labelleded with fluorescent-dye anti-porcine monoclonal antibodies for the following surface molecules: CD4 (clone 74–12-4, BD Pharmingen, CA, USA), CD8α (clone 76–2-11, BD Pharmingen, CA, USA), CD14 (clone TÜK4, Bio-Rad, CA, USA), CD16 (clone G7, Bio-Rad, CA, USA), CD27 (clone B30C7, Bio-Rad, CA, USA), CD45RA (clone MIL13, Bio-Rad, CA, USA), CD107a (clone 4E9/11, Bio-Rad, CA, USA), CD163 (clone 2A10/11, BD Pharmingen, CA, USA), and SLA-II (clone 2E9/13, Bio-Rad, CA, USA).

2 × 10^5^ cells were incubated for 30 min at 4 °C with adequate concentrations of monoclonal antibodies and the washed and re-suspended in PBS. The analysis was performed in a FACScalibur cytometer (BD Biosciences) after acquisition of 10^5^ events. First, cells were selected using forward and side scatter parameters, and then, were characterize by their fluorescence using CellQuest software (BD Biosciences, CA, USA). In all experiments, appropriate isotype-matched negative controls were included.

### Pericardial Fluid Analysis

Before intrapericardial administration of EV-CDCs and 24 h post-therapy, pericardial fluid samples were collected with an Abbocath*®-*T 20G catheter and then centrifuged for 5 min at 450 x g. The pellet was used for relative gene expression analysis with real time quantitative PCR (qPCR). Total RNA from pellets was purified using mirVANA miRNA isolation kit (Applied Biosystems, Foster City, CA), following the manufacturer’s protocol for total RNA extraction. Quality and concentration of total RNAs were evaluated by spectrophotometry. For each RNA sample, 1 μg of the corresponding cDNA was synthesized using iScript Reverse Transcription Supermix (BioRad, Hercules, CA, USA), according to manufacturer’s instructions. 1 μl of cDNA for each sample was then employed as template for the qPCR amplification with the TaqMan™ Fast Advanced Master Mix (Catalogue number 4444964, Thermo-Fisher Scientific Inc., MA, USA). Commercial TaqMan® Gene Expression Assays probes (Thermo-Fisher Scientific Inc., MA, USA) were used, according to manufacturer’s recommendations, to evaluate the relative expression of the following genes: IFN-γ (Ss03391054_m1), TNF (Ss03391318_g1), IL-2 (Ss03392428_m1), IL-12 (Ss03391176_m1), IL-4 (Ss03394125_m1), IL-5 (Ss03394369_m1), IL-10 (Ss03382372_u1), Arg1 (Ss03391394_m1), NOS2 (Ss03374608_u1), BPI (Ss04321426_m1) and CELA (Ss03392393_m1). Samples were evaluated in triplicate and 1 μl of water was substituted to templates to perform three negative controls for each probe. The qPCR reaction was performed in a QuantStudio 3 Real-Time PCR System (Applied Biosystems, Thermo Fisher Scientific Inc.), and the products were quantified by fluorescent method using 2^-ΔCt^ expression [21] with GAPDH (Ss03375629_u1) as endogenous control. All data were analyzed in the Thermo Fisher Cloud (also called Thermo Fisher Connect).

### Statistical Analysis

Data were statistically analyzed with SigmaPlot for Windows version 14 software (Systat Software, IL, USA). A Shapiro-Wilk test was used to assess normality. Paired comparisons were determined using a Student t-test for parametric data or a Wilcoxon signed rank test with the Yates continuity correction for non-parametric variables. qPCR data were analyzed using a Thermo Fisher Cloud Analysis version 1.0. Data are shown as mean ± standard deviation (SD). All *p*-values <0.05 were considered statistically significant.

## Results

Prior to animal studies, allogeneic CDCs from a single donor were isolated and characterized as previously described [16]. The dosage (30 × 10^6^ CDCs/animal) was selected on the basis of previous studies using allogeneic cardiac stem cells [16, 20]. Additionally, EV-CDCs were collected and isolated as previously described our group [18]. These vesicles showed a mean diameter of 198 nm and the proteomic analysis demonstrated a purity of 98% (percentage of proteins classified in the *Extracellular exosome* term by Gene Ontology) [22]. The supplementary fig. 1 shows the Field-Flow Fractionation and the classification of proteins by Gene Ontology. Moreover, according to MISEV2018 guidelines [23], our results demonstrated the expression of CD63, LAMP2, CD81 and CD9 molecules which are classified as “Transmembrane or GPI-anchored proteins associated to plasma membrane and/or endosomes”. Additionally, the proteomic analysis identified HSP90AB1 and HSPA1A proteins which are classified by MISEV2018 guidelines as “Cytosolic proteins recovered in EVs”.

In this study, CDCs and EV-CDCs were intrapericardially delivered in a closed chest porcine myocardial infarction model and the follow-up was constrained to the acute phase of myocardial infarction (Fig. 1). Cardiac function parameters and troponin I levels at 72 h after myocardial infarction evidenced that the myocardial infarction was successfully induced in all animals. The percentage of myocardial infarction ranged from 14% to 38% (21.93 ± 6.49) and left ventricular ejection fraction ranged from 20% to 45% (28.07 ± 6.08). It is important to note that, no significant difference was observed between randomized groups (Table 1).

**Table 1:** Data of cardiac function. Cardiac function parameters were determined 72 h after myocardial infarction induction in terms of: percentage of myocardial infarction (% Infarction), Left Ventricular Ejection Fraction (% LVEF), End Diastolic Volume index (EDVi), End Systolic Volume index (ESVi), heart rate and troponin I levels

Animal	% Infarction	% LVEF	EDVi	ESVi	Heart rate (bpm)	Troponin I (μg/l)
#1	28	25	79.8	60	103	5.5
#2	28	23	80.4	61.6	102	4.1
#3	18	28	69.7	50.4	89	8.9
#4	38	31	106.9	74.1	83	5
#5	19	25	105.89	79.66	100	1.5
#6	29	24	101.7	77.2	74	3.8
#7	26	23	75.9	58.8	81	3.2
#8	19	24	68.5	52	95	3.8
#9	18	33	89.9	60.3	68	7.5
#10	19	33	77.4	51.7	89	1
#11	18	29	74.6	52.6	85	0.6
#12	14	45	67.9	37.6	79	0.7
#13	20	20	73.9	59	88	6.3
#14	20	30	88.8	62.2	110	2.5
#15	15	28	99.9	72.1	89	1.8
Mean	21.93	28.07	84.08	60.62	89.00	3.75
SD	6.49	6.08	13.79	11.38	11.48	2.53

The in vivo monitoring was firstly focused on different biochemical parameters (Table 2). These biochemical parameters were determined before myocardial infarction (Basal) and 72 h after (Post-AMI). This analysis demonstrated that total proteins and urea were significantly reduced (Table 2). The analysis of biochemical parameters was also performed to compare the different study groups: Placebo, CDCs and EV-CDCs. In spite of the intrinsic variability between animals, the three groups showed an increase (although non-significant) in the GOT and GPT after treatments (Table 3).

**Table 2:** Biochemical parameters in basal conditions and after myocardial infarction induction. Blood samples were collected before acute myocardial infarction model creation (basal) and 72 h after (post-AMI). Normality was assessed using a Shapiro-Wilk test. Paired comparisons were performed using a Student t-test for parametric data or a Wilcoxon signed rank test with the Yates continuity correction for non-parametric variables. Values show the mean ± SD (n = 15). Numbers in bold show significant differences at p ≤ 0.05

	Basal (n = 15)	Post-AMI (n = 15)
Bilirubin (mg/dl)	0.35 **± 0.04**	**0.14 ± 0.04**
Creatinine (mg/dl)	1.65 ± 0.24	1.66 ± 0.24
GGT (U/l)	50.86 ± 7.07	39.80 ± 7.07
Glucose (mg/dl)	108.50 ± 12.83	88.40 ± 12.83
GOT (U/l)	**32.21 ± 19.18**	**56.80 ± 19.18**
GPT (U/l)	**31.00 ± 19.68**	75.80 **± 19.68**
Proteins (g/dl)	6.20 ± 0.39	5.22 ± 0.39
Urea (mg/l)	25.79 ± 4.62	22.42 ± 33.30

**Table 3.** Biochemical parameters after myocardial infarction induction and 24 h after treatment. Blood samples were collected 72 h after myocardial infarction model creation (post-AMI) and 24 h after intrapericardial administrations of Placebo, EV-CDCs and CDCs. Normality was assessed using a Shapiro-Wilk test. Paired comparisons were performed using a Student t-test for parametric data or a Wilcoxon signed rank test with the Yates continuity correction for non-parametric variables. Numbers in bold show significant differences (p ≤ 0.05) for each study group comparing post-AMI and 24 h after treatment. Table shows the mean ± SD (n = 5)

	PLACEBO GROUP		CDCs GROUP		EV-CDCs GROUP	
	Post-AMI (n = 5)	Post-therapy (n = 5)	Post-AMI (n = 5)	Post-therapy (n = 5)	Post-AMI (n = 5)	Post-therapy (n = 5)
Bilirubin (mg/dl)	0.19 ± 0.05	0.12 ± 0.05	0.14 ± 0.04	0.28 ± 0.30	0.13 ± 0.04	0.12 ± 0.03
Creatinine (mg/dl)	1.54 ± 0.34	1.48 ± 0.26	1.71 ± 0.19	1.52 ± 0.09	1.42 ± 0.30	1.46 ± 0.11
GGT (U/l)	41.80 ± 6.87	59.40 ± 21.52	36.00 ± 5.10	39.00 ± 5.10	44.00 ± 10.30	59.00 ± 22.73
Glucose (mg/dl)	93.60 ± 19.89	95.20 ± 20.97	86.20 ± 9.60	109.75 ± 16.11	78.20 ± 19.07	89.00 ± 23.82
GOT (U/l)	**61.00 ± 22.12**	**95.00 ± 13.17**	54.60 ± 20.27	116.50 ± 71.59	47.60 ± 17.62	89.40 ± 43.47
GPT (U/l)	75.00 ± 25.81	83.00 ± 20.94	70.20 ± 19.75	88.00 ± 19.61	68.60 ± 21.98	74.20 ± 14.29
Proteins (g/dl)	**5.33 ± 0.45**	**6.35 ± 0.52**	5.24 ± 0.37	5.61 ± 0.40	5.60 ± 0.30	6.51 ± 0.36
Urea (mg/l)	21.56 ± 5.66	28.14 ± 3.95	21.78 ± 7.61	24.46 ± 8.85	21.78 ± 7.61	24.46 ± 8.85

**Fig. 2:**
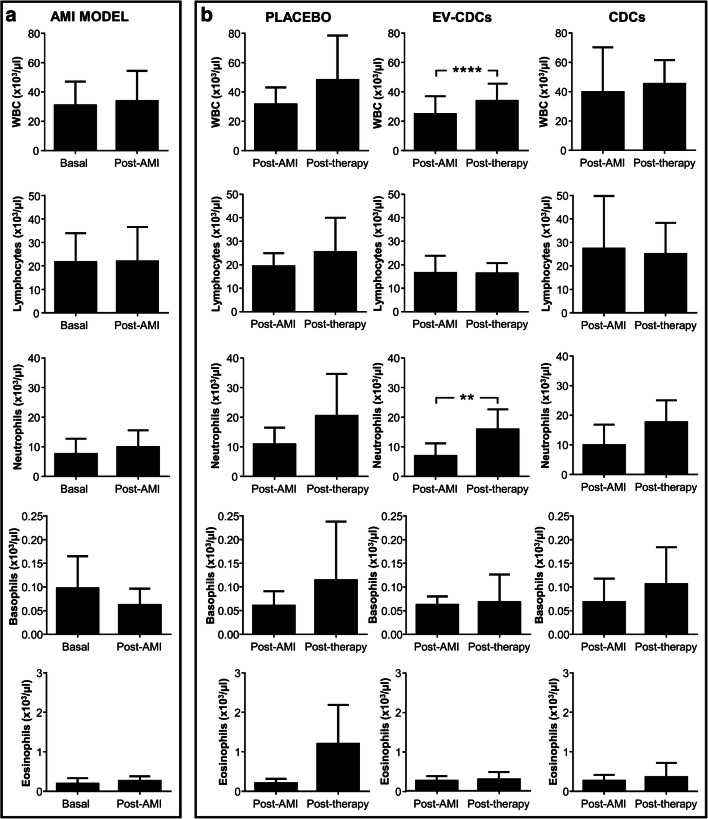
White blood cell analysis in peripheral blood. Blood samples were collected in EDTA containing tubes before acute myocardial infarction model creation (Basal), 72 h after (Post-AMI) and 24 h after the treatment (Post-therapy) and white blood cells were counted in an automated hematology analyzer. Normality was assessed using a Shapiro-Wilk test. Paired comparisons of the AMI model (**a**)(*n* = 15) and paired comparisons of the administered therapies (**b**) (*n* = 5) were performed using a Student t-test for parametric data or a Wilcoxon signed rank test with the Yates continuity correction for non-parametric variables. Graphs show the mean ± SD of cell populations. **p ≤ 0.01. ****p ≤ 0.0001

Our determinations also included the quantification of hematological parameters. The hematological analysis before myocardial infarction and 72 h after did not show any significant difference (Fig. 2a). However, White Blood Cells (WBC) and neutrophils were significantly increased after EV-CDC treatment (Fig. 2b). Taking into account that WBC quantification is the sum of all leukocytes, the increase of WBC is reflecting this neutrophils increase.

Apart from the biochemical and hematological analyses, the main goal of this study was to perform a deep characterization of peripheral blood lymphocyte subsets to determine the hypothetical immunomodulatory effect of these therapies. Although the scarce commercial availability of reagents for the porcine model is a limiting factor in these studies, here we could quantify CD4+ T cells (also called helper T cells), CD8+ T cells (also called cytotoxic T cells), NK cells (here defined as CD4-/CD16+) and double positive cells (CD4+/CD8+), which are considered by other authors as T helper memory cells [24, 25]. Additionally, the analysis of CD4+ T cells and CD8+ T cells was also focused on their differentiation/activation status using CD27 and CD45RA markers. The co-expression analysis of these two markers allowed us to identify naïve T cells (CD27+ CD45RA+) and effector/memory T cells (CD27- CD45RA-). Not only peripheral blood lymphocytes were analyzed, but also monocyte counts, as well as the percentage of circulating M2 monocytes (here defined as CD14 + CD163+).

**Fig. 3:**
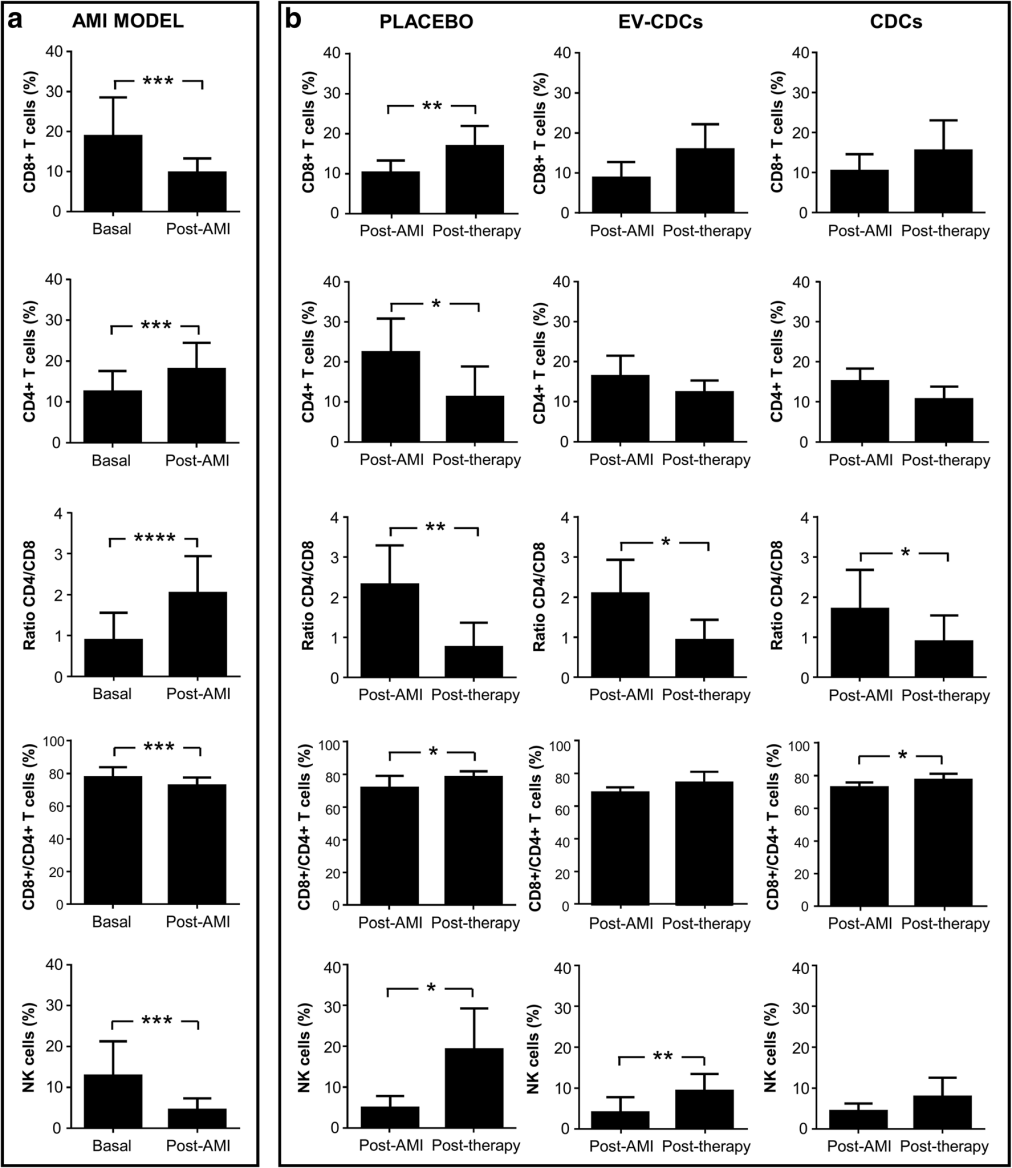
Lymphocyte subsets distribution in peripheral blood. Peripheral blood lymphocytes were isolated from blood samples before acute myocardial infarction model creation (Basal), 72 h after (Post-AMI) and 24 h after the treatment (Post-therapy). Lymphocyte subsets distribution was analyzed by flow cytometry. Normality was assessed using a Shapiro-Wilk test. Paired comparisons of the AMI model (**a**)(n = 15) and paired comparisons of the administered therapies (**b**) (n = 5) were performed using a Student t-test for parametric data or a Wilcoxon signed rank test with the Yates continuity correction for non-parametric variables. Graphs show the mean ± SD of cell populations. *p ≤ 0.05. **p ≤ 0.01. ***p ≤ 0.001. ****p ≤ 0.0001

As expected, significant changes were observed when compared T cell subsets before myocardial infarction and 72 h after. The CD4/CD8 ratio was significantly higher, as a consequence of CD8 + T cells decrease, and CD4+ T cells increase (Fig. 3a).

Not only CD4 + T cells and CD8+ T cells were altered after myocardial infarction. In our lymphocyte analysis, the CD4+/CD8+ double positive cells and NK cells were also significantly decreased after myocardial infarction (Fig. 3a). Unfortunately, the analysis of these lymphocyte subsets at 24 h post-therapy did not show any relevant difference in the different study groups. Uniquely, the CD4/CD8 ratio was significantly reduced in all groups when compared to post-AMI.

**Fig. 4:**
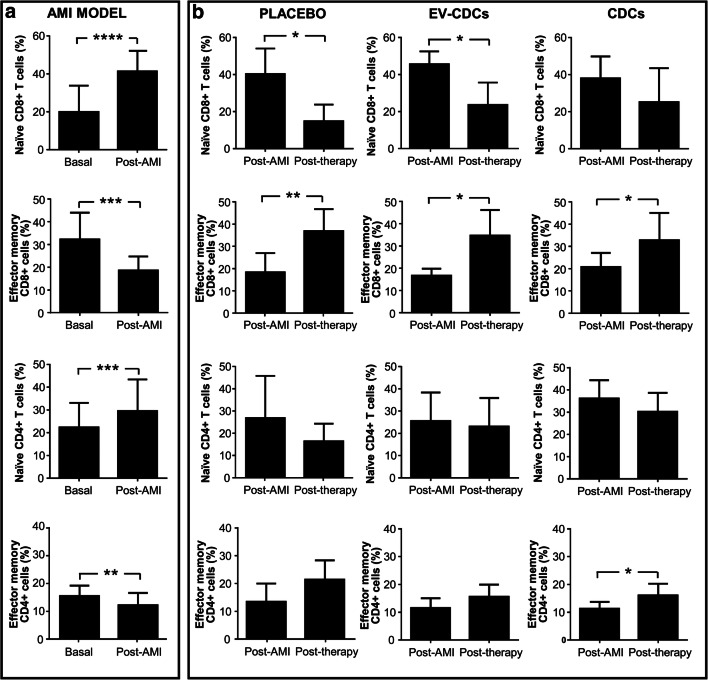
Differentiation/activation T cell subsets status in peripheral blood. Peripheral blood lymphocytes were isolated from blood samples before acute myocardial infarction model creation (Basal), 72 h after (Post-AMI) and 24 h after the treatment (Post-therapy). T cell subset status was analyzed by flow cytometry using CD27 and CD45RA markers. The co-expression analysis of these two markers allowed us to identify naïve T cells (CD27+ CD45RA+) and effector/memory T cells (CD27- CD45RA-). Normality was assessed using a Shapiro-Wilk test. Paired comparisons of the AMI model (**a**) (n = 15) and paired comparisons of the administered therapies (**b**) (n = 5) were performed using a Student t-test for parametric data or a Wilcoxon signed rank test with the Yates continuity correction for non-parametric variables. Graphs show the mean ± SD of cell populations. *p ≤ 0.05. **p ≤ 0.01. ***p ≤ 0.001. ****p ≤ 0.0001

In the comparative analysis of naïve and effector memory cells (performed in CD4 + T cells and CD8+ T cells) significant differences were found when compared basal and post-AMI (Fig. 4a). These changes were reverted after placebo, EV-CDCs and CDCs treatments (Fig. 4b).

**Fig. 5:**
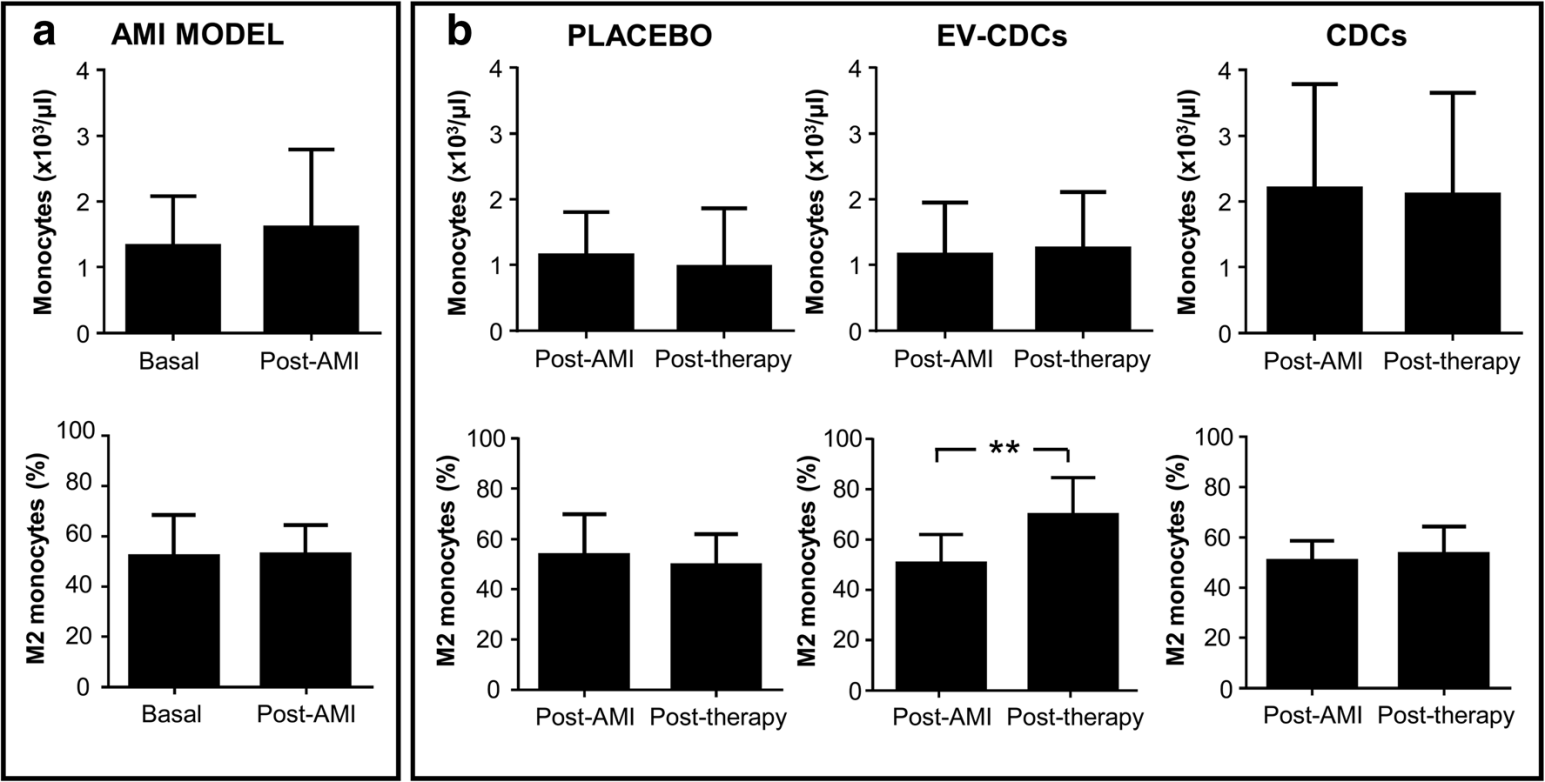
Monocyte populations in peripheral blood. Blood samples were collected in EDTA containing tubes before acute myocardial infarction model creation (Basal), 72 h after (Post-AMI) and 24 h after the treatment (Post-therapy). Monocyte count was performed in an automated hematology analyzer and its phenotype characterization was evaluated by flow cytometry, defining circulating M2 monocytes as CD14 + CD163+. Normality was assessed using a Shapiro-Wilk test. Paired comparisons of the AMI model (**a**)(n = 15) and paired comparisons of the administered therapies (**b**) (n = 5) were performed using a Student t-test for parametric data or a Wilcoxon signed rank test with the Yates continuity correction for non-parametric variables. Graphs show the mean ± SD of cell populations. **p ≤ 0.01

The multiparametric flow cytometry analysis was finally focused in the percentage of circulating CD14 + CD163+ cells. Our results demonstrated that EV-CDCs treatment significantly increased the percentage of these cells in peripheral blood (Fig. 5b). This difference was not observed in the placebo, or in the CDCs group and any significant difference was found in terms of monocyte counts.

**Fig. 6:**
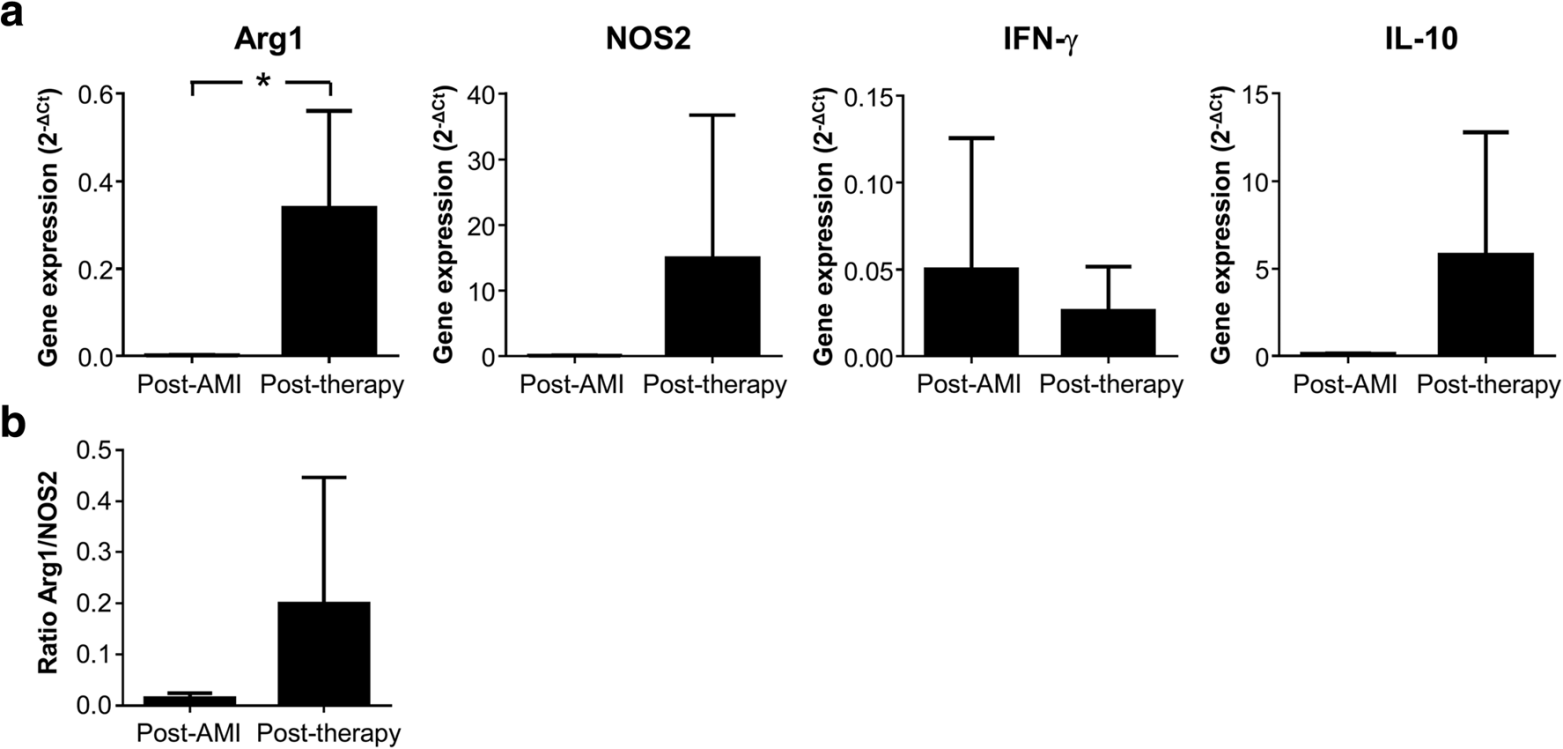
Cytokines gene expression in pericardial fluid cells. Pericardial fluids were compiled before and 24 h after EV-CDCs administration. Total RNA was isolated from pericardial fluid cells and qPCR products were quantified by the 2^-∆Ct^ method using GAPDH as an endogenous control. The statistical analysis was performed using a Thermo Fisher Cloud Analysis version 1.0. Graphs show the mean ± SD (*n* = 3). *p ≤ 0.05

In order to extend our results from peripheral blood to heart tissue, pericardial fluids were collected to determine the inflammatory environment before and after EV-CDCs administration. A Real-time quantitative PCR was performed to quantify the expression of TH1 cytokines (IFN-γ, TNF-α, IL-2 and IL-12), TH2 cytokines (IL-4, IL-5 and IL-10), M1/M2 markers (Arg1 and NOS2), as well as neutrophils markers (BPI and CELA). Arg1, NOS2, IFN-γ, and IL-10 were successfully amplified and detected in all individuals, showing a significant increase of Arg1 at 24 h post-therapy, as well as a non-significant increase of NOS2, IFN-γ, and IL-10 (Fig. 6a). Unfortunately, the cDNA amplification was unsuccessful for the other TH1 and TH2 cytokines, probably due to the limited amount of pericardial leukocytes. Arg1 is a classical M2 marker for monocytes [26] and neutrophils [27], so the increase of Arg1/NOS2 ratio after EV-CDCs administration (Fig. 6b) confirmed the M2 polarization observed by flow cytometry.

## Discussion

There is extensive literature describing the therapeutic potential of CDCs in myocardial infarction [28], which has been extended to other diseases such as Duchenne muscular dystrophy [29] and aging [14]. Nowadays, although the therapeutic effect of CDCs in aging mouse hearts is a matter of debate [30, 31], it is widely accepted that CDCs attenuate the inflammation during myocardial injury [7, 8, 16]. In vitro studies using CDCs have also demonstrated that the anti-inflammatory, anti-apoptotic, and pro-angiogenic effects of are mediated, at least in part, through the release of extracellular vesicles [32–34]. These CDCs-derived exosomes have been recently evaluated in a clinically relevant animal model of myocardial infarction demonstrating a therapeutic effect in adverse remodeling and scarring [11]. This therapeutic effect was solely observed after intramyocardial delivery while the intracoronary administration was ineffective and very similar to placebo.

In this study, we aimed to evaluate the immunomodulatory effect of intrapericardially delivered CDCs and EV-CDCs in a clinically relevant myocardial infarction model. The intrapericardial delivery is considered a safe and effective route for stem cell-based therapies [15, 16]. Our group has previously demonstrated that, the pericardial fluid preserve the viability of BM-MSCs favoring the adhesion and homogeneous distribution of administered cells without adverse effects [15]. Moreover, the intrapericardial administration of CDCs altered immunological parameters in a myocardial infarction model [16]. An important advantage of using our animal model (a close chest experimental porcine model of myocardial infarction), is the repeatability and homogeneity of study groups. However, this fact could also be considered a drawback as myocardial infarction in humans is frequently related with other complications.

In our preclinical study, we demonstrated that the intrapericardial administration of 30 × 10^6^ CDCs/animal was simple and safe, which is in agreement with our previous studies [15]. Similarly, any adverse reaction was observed after EV-CDCs administration. In the follow-up period, our first set of determinations was focused on the quantification of biochemical parameters. The three groups showed a non-significant increase of GOT and GPT at 24 h after treatments which is in agreement with our own findings [35]. The alterations of biochemical parameters during cardiac failure is a very common event and associated with a hepatic dysfunction due to an increase in hepatic veins pressure [36]. Moreover, surgical approaches such as laparoscopy could also increase serum transaminase levels [37].

In the hematological analysis, our results showed a significant increase in neutrophils after EV-CDC treatment. It is well known that neutrophils are recruited into the infarcted area during the inflammatory phase, being attracted by cell debris and inflammatory signals. These cells have been usually considered as pro-inflammatory cells and according to clinical outcomes in myocardial disease, the increase of circulating neutrophils after EV-CDCs administration could be correlated with an aggravated outcome [38]. On the contrary, there are evidences for the pro-regenerative and anti-inflammatory capacity of these cells in myocardial infarction [27, 39]. Considering this duality, the increase of circulating neutrophils after EV-CDCs treatment deserves further investigation and a full characterization of these neutrophils.

In order to determine the hypothetical immunomodulatory effect of these therapies, peripheral blood lymphocytes were characterized by multiparametric flow cytometry. In this analysis, the CD4/CD8 ratio was significantly reduced in all groups when compared to post-AMI. Most probably, this change could be the consequence of leukocytes redistribution from blood to the inflammation site or homeostasis-restoring mechanisms to basal conditions. Our second hypothesis is that, the surgical approach itself may initiate a post-operative immunosuppression. Supporting this idea, a previous study comparing laparoscopy and thoracotomy approaches demonstrated that CD4 + T cells and lymphocyte numbers were significantly reduced in the thoracotomy group, suggesting a post-operative immunosuppression after thoracotomy approach [40].

The immunomodulatory analysis of CDCs and EV-CDCs therapies was completed with the quantification of CD14 + CD16+ cells in peripheral blood. These cells correspond to pro-angiogenic and immunomodulatory subsets of monocytes (also defined as “M2 monocytes”) [41]. Taking into account that M2 polarization contributes to resolution of inflammation promoting tissue repair [42–44], here we hypothesize that the increase of M2 monocytes after EV-CDCs treatment might counteract the exacerbated inflammatory response in the acute phase of myocardial infarction. A similar hypothesis was proposed by Sekerkova et al., where these cells “might play a protective role in the early phase after kidney transplantation” [45]. It is important to note that the increase of M2 monocytes was solely observed in EV-CDCs, but not in CDCs. Taking into account that, the concentration of paracrine factors released by 30 × 10^6^ allogeneic CDCs must be significantly lower than the average of paracrine factors in EV-CDCs, we consider that there may be a dose-response relationship between these factors and M2 differentiation.

A significant and innovative aspect of our study lies in the use of a different administration route. In this sense, and in agreement with de Couto et al. [12], our results have demonstrated the immunomodulatory effects of EV-CDCs in a clinically relevant animal model of myocardial infarction. While previous in vivo experiments have been performed by open-chest intramyocardial injection, here we demonstrate that intrapericardial administration was a safe and efficient alternative for reducing the adverse or unbalanced inflammatory reaction triggering the M2 polarization in circulating monocytes and pericardial leukocytes.

Although it was not the purpose of the study, the long-term effect of these treatments (in terms of cardiac functionality) could be determined at 10 weeks. Unfortunately these results were not conclusive, more especially since these healthy and young animal models are characterized by an early regenerative potential which could mask the therapeutic effect of the treatments [46]. In any case, our results did not show any significant difference between groups (Ejection Fraction: 29.6 ± 8.0 in Placebo, 29.8 ± 17.2 in EV-CDCs, 32.0 ± 6.7 in CDCs; % Infarct: 12.4 ± 3.5 in Placebo, 10.6 ± 1.7 in EV-CDCs, 9.2 ± 4.0 in CDCs).

Obviously, one of the limitations of the study is related to the identification of molecular mechanisms involved in M2 polarization. The group of E. Marban has already demonstrated that Y RNA fragment [47] and miR-181b [12] are directly implicated in the immunomodulatory effect of EV-CDCs. In this sense, our research group is currently focused in “OMICS” studies, analyzing miRNA profiles by Next Generation Sequencing and proteins by high throughput proteomic analysis. Our preliminary results from these studies are showing an abundant expression of immune-related proteins and miRNAs (manuscript in preparation).

In conclusion, this is the first report where stem cell-derived extracellular vesicles have been intrapericardially administered in a clinically relevant animal model of myocardial infarction. In our experimental conditions and dose, EV-CDCs stimulate a M2 polarization during the acute phase of porcine myocardial infarction. Moreover, the immunomodulatory effects of EV-CDCs, after intrapericardial administration, were comparable to previous studies using the open-chest intramyocardial injections. Finally, the intrapericardial administration route offers the possibility of a minimally invasive surgical approach and could be more advantageous from a clinical perspective.

## Electronic supplementary material

Supplementary fig.1 Characterization of EV-CDCs by flow field-flow fractionation (A) and proteomic analysis (B). Field-Flow Fractionation was carried out with a regenerated cellulose membrane (cut off 10 kDa) and with a spacer of 350 mm. PBS (filtered through 0.1 mm Durapore membrane) was used as the carrier. Diameter (nm): 198. Estimated NW (kDa): 1,56*105 (A). Peptide and scan counting was performed assuming as positive events those with a FDR equal or lower than 5%. This proteomic analysis allowed us to identify a total of 759 proteins (with more than two peptides per protein at 1% FDR). Proteins were classified according to Gene Ontology term GO:0070062 (B). (PNG 629 kb)

High Resolution Image (TIF 250 kb)
