# Supplementary figures and images for: The Intrapericardial Delivery of Extracellular Vesicles from Cardiosphere-Derived Cells Stimulates M2 Polarization during the Acute Phase of Porcine Myocardial Infarction
Source: Stem Cell Rev Rep. 2019 Dec 21;16(3):612–25. doi: 10.1007/s12015-019-09926-y (PMC7253530; doi:10.1007/s12015-019-09926-y)

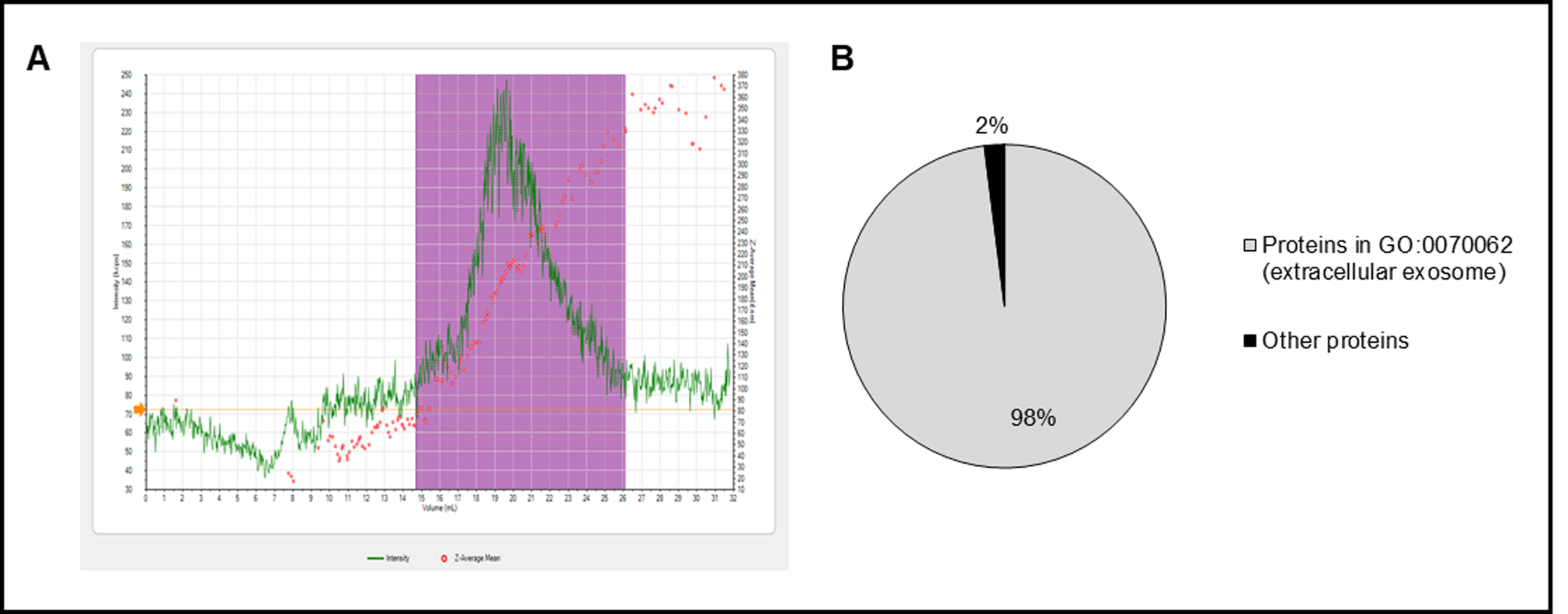

Supplement: Supplementary file 1 — Characterization of EV-CDCs by flow field-flow fractionation (A) and proteomic analysis (B). Field-Flow Fractionation was carried out with a regenerated cellulose membrane (cut off 10 kDa) and with a spacer of 350 mm. PBS (filtered through 0.1 mm Durapore membrane) was used as the carrier. Diameter (nm): 198. Estimated NW (kDa): 1,56*105 (A). Peptide and scan counting was performed assuming as positive events those with a FDR equal or lower than 5%. This proteomic analysis allowed us to identify a total of 759 proteins (with more than two peptides per protein at 1% FDR). Proteins were classified according to Gene Ontology term GO:0070062 (B). (PNG 629 kb) [file 12015_2019_9926_Fig7_ESM.png]

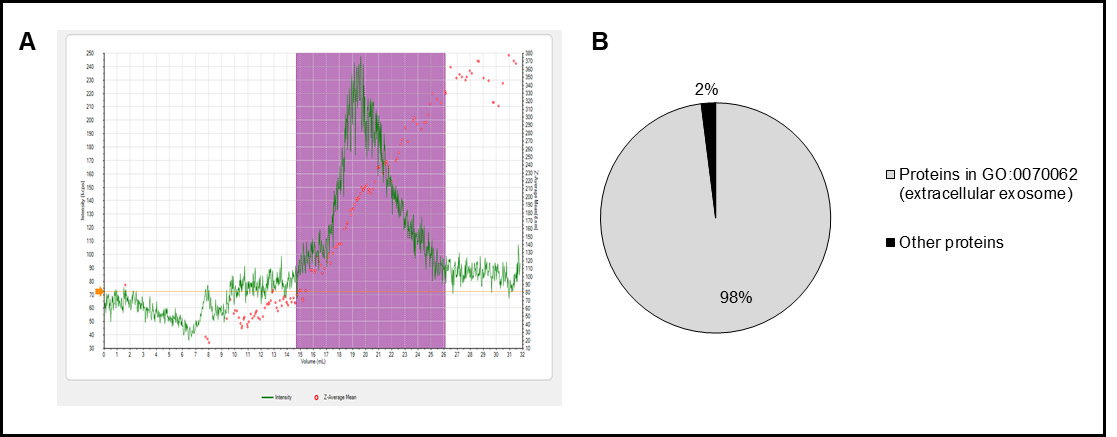

Supplement: Supplementary file 2 — High Resolution Image (TIF 250 kb) [file 12015_2019_9926_MOESM1_ESM.tif]
